# Impact of Particle and Equipment Properties on Residence Time Distribution of Pharmaceutical Excipients in Rotary Tablet Presses

**DOI:** 10.3390/pharmaceutics12030283

**Published:** 2020-03-21

**Authors:** Daniel Puckhaber, Sebastian Eichler, Arno Kwade, Jan Henrik Finke

**Affiliations:** 1 Institute for Particle Technology, Technische Universität Braunschweig, Volkmaroder Str. 5, 38104 Braunschweig, Germany; a.kwade@tu-braunschweig.de (A.K.); jfinke@tu-braunschweig.de (J.H.F.); 2 Center of Pharmaceutical Engineering (PVZ), Technische Universität Braunschweig, Franz-Liszt-Str. 35A, 38106 Braunschweig, Germany; 3 KORSCH AG, Breitenbachstr. 1, 13509 Berlin, Germany; sebastian.eichler@korsch.de

**Keywords:** tableting, powder flow, feed frame, scale-up, intermixing

## Abstract

Paddle feeders are devices commonly used in rotary tablet presses to facilitate constant and efficient die filling. Adversely, the shear stress applied by the rotating paddles is known to affect the bulk properties of the processed powder dependent on the residence time. This study focuses on the residence time distribution (RTD) of two commonly applied excipients (microcrystalline cellulose, MCC; dicalcium phosphate, DCP), which exhibit different flow properties inside rotary tablet presses. To realistically depict the powder flow inside rotary tablet presses, custom-made tracer powder was developed. The applied method was proven to be appropriate as the tracer and bulk powder showed comparable properties. The RTDs of both materials were examined in two differently scaled rotary tablet presses and the influence of process parameters was determined. To analyze RTDs independent of the mass flow, the normalized variance was used to quantify intermixing. Substantial differences between both materials and tablet presses were found. Broader RTDs were measured for the poorer flowing MCC as well as for the production scale press. The obtained results can be used to improve the general understanding of powder flow inside rotary tablet presses and amplify scale-up and continuous production process development.

## 1. Introduction

Tablets are the most commonly administered solid dosage form and often the dosage form of choice during the development of new drugs due to their beneficial properties for both patients (easy and convenient application, good dosability) and manufacturer (storage stability, economical production). The economical production of large tablet quantities in industrial pharmacy is facilitated by employing rotary tablet presses which enable tablet production rates up to 1,000,000 tablets per hour.

### 1.1. Powder Flow in Rotary Tablet Presses

The production capacity of a given tablet press and tooling is directly determined by the rotation frequency of the die table and limited by the die filling step [1,2,3,4]. An incomplete or inconsistent die filling cannot be compensated in subsequent process steps and results in inhomogeneities of tablet mass, drug content and, for constant compression heights, insufficient mechanical strength. Thus, the die filling step can be regarded as a critical step determining the final quality-defining tablet properties and complete die filling has to be assured even at the highest production rates [1,4,5]. The powder flow into the dies generally depends on both the bulk powder properties and process setup [6,7,8]. Typically, the powder properties of a given formulation are preliminary designed and fixed, limiting the adjustable parameter to improve powder flow to the process setup and particularly, the powder feeding system of the tablet press. Powder feeding systems of rotary tablet presses typically consist of a hopper connected to a feed frame which is equipped with at least one rotating paddle which forces the powder to flow towards the withdrawal area inside the feed frame [9,10]. In principle, feed frames are devices to increase the powder flow rates inside the tablet press and ensure a high die filling efficiency and consistency to enable high production capacities. However, the powder flow through the tablet press in general and the feed frame especially is known to possibly cause several unwanted changes of the powder blend properties, which result in an alteration of tablet-quality defining attributes, e.g., mechanical strength. There have been several recent studies regarding the effect of the feed frame on bulk properties, both experimental [11,12,13,14] as well as computational studies [15,16,17]. They identified various mechanisms negatively affecting the bulk properties, including overlubrication and abrasion caused by stresses in the feed frame, as well as segregation of blends. Mateo-Ortiz et al. numerically investigated possible particle attrition due to the stress applied by the paddles within the feed frame and found rising tangential stress for higher paddle speeds [16]. These findings were in good accordance with results obtained by Mendez et al. who found altered particle size distribution after the feed frame passage [14]. As these forces act continuously over time, particles that stay longer in the feed frame are more likely to be affected. A comparable influence of residence time on the change in powder properties was observed by Narang et al. who found a reduced mechanical strength for a lubricated model formulation for longer residence times in the feed frame due to the continuously progressing dispersion of the lubricant [13]. These two publications show the clear influence of the different residence times in the filling system on the changes in powder properties and illustrate the high relevance of residence time investigations for a better understanding of influencing factors.

### 1.2. Continuous Manufacturing in Pharmaceutical Industry

Additionally, continuous production processes are currently of great interest in the pharmaceutical industry as they offer several potential economic advantages, such as an improved footprint, higher agility, and flexibility during production and simplified scalability [18,19,20]. Continuous production processes require the application of in-line measurements to ensure that the product meets the quality attributes and to initiate countermeasures if quality attributes are out of specification [19,20,21,22]. To successfully utilize in-line data, an improved understanding of the underlying dependencies between process parameters and process conditions is needed. In the case of tableting, the powder flow behavior dictates the quality attributes and thus, the influence of material and process parameters on the powder flow behavior has to be investigated. One possibility to investigate the powder flow in process devices is the determination of residence time distributions (RTDs) [23,24,25,26]. The determination of RTDs of powder within tablet presses can be used to improve the fundamental understanding of its flow behavior as well as influencing factors and thus, facilitate the implementation of continuous tableting production processes.

### 1.3. Residence Time Distribution in Tablet Production

The RTD of the processed powder and the impact of process and bulk properties on it are of great interest to predict and avoid unwanted powder property changes as well as to facilitate the continuous production of pharmaceutical products. The correlation between the powder behavior inside the tablet press and the applied process parameters such as turret speed, paddle speed, or paddle geometry are particularly relevant. In recent years, there has been a rising number of studies dealing with the determination of RTDs of traced powder inside the feed frame of rotary tablet presses [11,16,27,28,29,30]. In addition to those experimental studies, some researchers have conducted simulations of the residence time distribution inside tablet presses using the discrete element method [16,31]. Dülle et al. have conducted an extensive series of experiments investigating the effect of applied tracer amount, powder flow rate, moisture content, tracer particle size [27], scraper position, turret speed, filling height, and paddle speed [28], as well as differences in feed frame design (e.g., paddle geometry or insert of a volume-reducing perspex disc) [29,30] on RTDs of dyed microcrystalline cellulose (MCC) in the feed frame (Fill-O-Matic) of a Fette 102i. Besides other findings, they showed a considerable decrease in the mean residence time for higher turret speeds as well as narrower RTDs for slower paddle speeds. On the contrary, Mateo-Ortiz et al. observed narrower RTDs when using higher paddle speeds. This difference is probably due to the use of a tracer which had significantly different properties than the bulk material [16]. However, extensive studies for other materials than MCC are not available until today and the transferability of the published results to other rotary tablet presses as well as other commonly used excipients exhibiting different flow behavior is unknown. This calls for an increased need for further investigation of the impact of process parameters on the RTDs for bulk powder with different powder flow behavior by applying particulate tracer materials which realistically represent the particulate properties of the investigated excipients. The investigation of the residence time behavior of pharmaceutical excipients can help to improve the general understanding of the influence of process and formulation parameters on powder flow behavior inside tablet presses. 

This work aims to develop a method to produce particulate tracer which exhibits particle and bulk properties such as powder flow properties similar to commonly used excipients for solid dosage forms. The produced tracer powder is used to investigate the impact of process parameters as turret speed, paddle speed, and paddle geometry on the RTD of two commonly applied excipients on differently scaled rotary tablet presses. Obtained insights should be used to improve the general knowledge of the powder flow behavior of pharmaceutical powder inside rotary tablet presses to facilitate the further development of continuous tableting processes.

## 2. Materials and Methods 

### 2.1. Materials

Microcrystalline cellulose (MCC, Vivapur 102®) and anhydrous calcium hydrogen phosphate (DCP; EMCOMPRESS® Anhydrous; both J. Rettenmaier & Söhne GmbH &Co. KG, Rosenberg, Germany) were used as model materials for this study. As a lubricant, 1.5 wt% magnesium stearate (MgSt, MAGNESIA GmbH, Lüneburg, Germany) was added to DCP for all purposes. For the production of the tracer powder, methylene blue (Sigma Aldrich, Darmstadt, Germany) was applied as a dye.

### 2.2. Methods

#### 2.2.1. Production of Tracer Powder

Tracer powders were produced by dyeing 100 g of MCC or DCP with 50 mL of a 10 mM aqueous methylene blue solution in a fluidized bed granulator (Mini-Glatt, Glatt GmbH, Binzen, Germany) in top-spray configuration. The methylene blue solution was sprayed using a peristaltic pump with a volumetric flow of 2 mL min^−1^ using a nozzle with a diameter of 0.5 mm. After the dyeing process was completed, the dyed powder was dried for 10 min by continuing the fluidization process at 80 °C.

#### 2.2.2. Analysis of Particle Size Distribution

The particle size distributions of bulk and tracer powders were analyzed using dynamic image analysis (QicPic, Sympatec GmbH, Clausthal, Germany) using the GRADIS dry powder dispersion unit. Particle sizes were obtained by calculating the diameter of a circle with an equal projection area. For each sample, at least 100,000 particles were analyzed.

#### 2.2.3. Determination of Bulk and Tapped Density

Bulk densities *ρ_b_* of excipients and tracer powders were examined by carefully pouring powder with known mass into a 250 mL cylinder and measuring the occupied volume. Afterward, the powder was tapped by a volumetric analyzer (SVM 121, ERWEKA GmbH, Langen, Germany) with 1250 movements and the tapped density *ρ_T_* was calculated. The ratio of bulk and tapped density is known as the Hausner ratio *HR*, which describes the sensitivity of a powder to be compressed by gravitational forces and can be used to roughly estimate the interparticulate interactions and thereby, roughly assess the powder flow behavior:

(1) $$HR=\frac{\rho_{T}}{\rho_{b}}$$

The obtained *HR*-values can be used to categorize the predicted powder flow behavior according to Carr [32].

#### 2.2.4. Ring Shear Cell Measurements

The powder flowability of both excipients and tracer powders were examined by using a ring shear cell tester (RST-XS, Dr. Dietmar Schulze Schüttgutmesstechnik, Wolfenbüttel, Germany) and applying normal stresses of 2.5, 5, 10, and 20 kPa with five sampling points, respectively. The yield strength *σ_c_* for a given consolidation stress *σ*_1_ was measured to calculate the flowability *ffc*:

(2) $$ff_{c}=\frac{\sigma_{1}}{\sigma_{c}}$$

The resulting *ffc* values can be used to categorize the predicted powder flow behavior according to the classification of Jenike [33].

#### 2.2.5. Experimental Setup of Residence Time Experiments

The RTDs of MCC and DCP were determined by introducing a tracer pulse into the powder feeding system of two different rotary tablet presses. As rotary tablet presses, a pilot-scale XL 100 and a production scale XL 400 (both KORSCH AG, Berlin, Germany) were examined. For all experiments at the XL 100, 10 g tracer powder was introduced as an even layer at the lower end of the inspection glass inside the feeding pipe which is mounted directly on top of the feed frame. Due to the different construction of the feed frame at the XL 400, 30 g tracer powder was introduced at the highest entry point of the feed frame. As a consequence of the different structures of both feed frames, the investigated volume vastly differed (XL 100: 398 cm^3^; XL 400: 913.1 cm^3^). After introducing the tracer powder, this layer was again covered with the respective bulk material.

**Figure 1:**
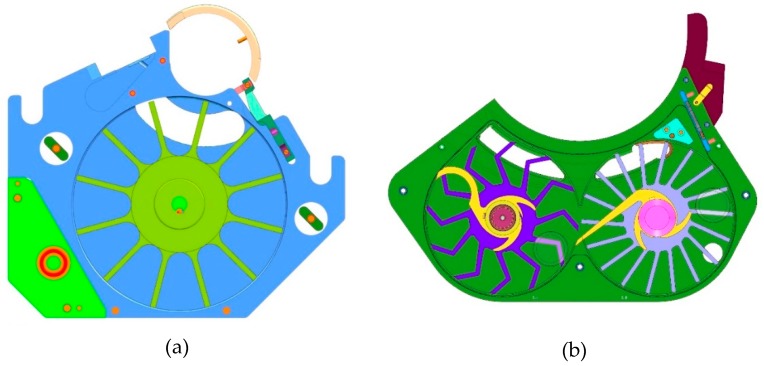
Feed frame of XL 100 (**a**) exhibiting a single chamber equipped with one dosing paddle and feed frame of XL 400 (**b**) consisting of a dosing chamber (left) and metering chamber (right) equipped with a dosing and metering paddle, respectively.

The XL 100 was equipped with a single chamber feed frame employing one dosing paddle, while the feed frame of the XL 400 consisted of two chambers: a dosing chamber which feeds the powder into the dies using a dosing paddle and a counter-rotating metering paddle in the metering chamber which transfers the overdosed powder back into the feed frame (Figure 1).

**Figure 2:**
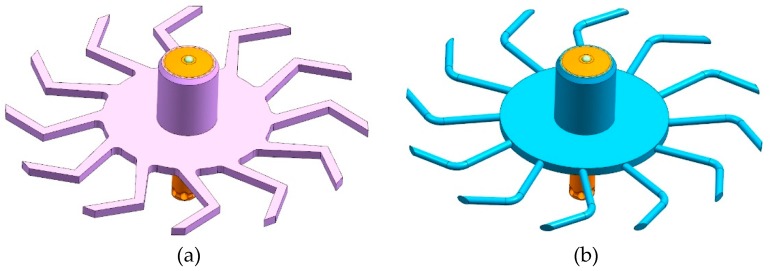
Dosing paddle geometries of XL 400: (**a**) rectangular, curved bars; (**b**) round, curved bars.

Depending on the applied tablet press, process parameters were systematically varied (Table 1). A detailed overview of the different applied process parameter combinations is shown in the Supplementary Material (Table S1). The influence of the dosing paddle shape on the RTD of DCP at the XL 400 was evaluated by applying two different geometries (Figure 2). The clearance between bars of the dosing paddle and the bottom plate of the feed frame differed for both applied geometries. The dosing paddle with rectangular, curved bars exhibited a clearance of 0.7 mm and 1 mm for the round, curved bars to the bottom plate, respectively.

To produce tablets with a comparable target mass of 250 ± 20 mg during all experiments, tailored filling and dosing heights were applied (Table 2).

For all experiments performed at the XL 100, a die table equipped with four flat-faced, round 9 mm Euro-D tooling sets was used. The transferability of obtained results to a production press was examined by using a die table equipped with 29 flat-faced, round 7 mm Euro-D tooling sets at the XL 400. For all experiments, the rotary tablet press was filled with bulk powder and tablets were produced until a stable process was attained, indicated by a constant compaction force. Afterward, the tracer powder was introduced as a layer (XL 100: 10 g; XL 400: 30 g). Subsequently, the bulk powder was added on top of the tracer layer until the feeding system of the rotary tablet press was filled. Tablets were continuously produced and samples consisting of three tablets were taken at 24 different points of time. The time interval between samples was selected according to the necessary time for the tracer to be transported through the tablet press. The run time depended on the process parameters and is listed in the Supplementary Material (Table S2). The compaction pressure was chosen to ensure both tablet integrity during storage and grindability during analysis. 

#### 2.2.6. Determination of Methylene Blue Concentration

Methylene blue concentration of sampled tablets was measured by UV-Vis spectroscopy (UV-3100 PC, VWR International GmbH, Darmstadt, Germany) using a previously conducted linear calibration linking methylene blue concentration and absorption. Samples consisting of three tablets were ground with a pestle and mortar. Afterward, 100 mg of the ground powder was dispersed in 10 mL of a 1 M aqueous hydrochloric acid solution and stirred for 10 s using a vortex blender. 3 mL of the supernatant was filtered through a microporous filter (mean pore size of 0.45 µm) to remove residual particles. The filtered solution was photometrically examined at a wavelength of 660 nm as a three-fold determination and the average methylene blue concentration was calculated. Measured absorbance values below 0.005 were considered 0 as they were outside the calibration range. To correct the possible adsorption of solved tracer on particle surfaces, an additional calibration was performed. A linear relationship between methylene blue concentration of pure tracer and blends of tracer and bulk powder (applying a constant amount of tracer powder) was found and applied as a correction factor. 

#### 2.2.7. Evaluation of RTDs

**Figure 3:**
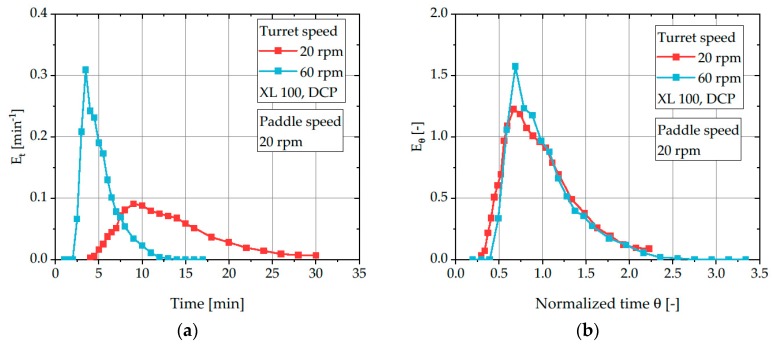
Residence time distribution *E_t_* (**a**) and normalized residence time distribution *E_θ_* (**b**) for different turret speeds using DCP as a tracer and bulk powder at the XL 100.

At each point in time *t*, three tablets were sampled and the methylene blue concentration *c(t)* was measured to create a time versus concentration profile, called the residence time function *E_t_* (Figure 3a). The fundamentals of residence time experiments were first introduced by Danckwerts [34]. A useful overview of the derived characteristics of RTDs used in this study can be found in Naumann [35]. In general, the residence time function describes the percentage of tracer particles which left the system in a given time step *dt*:

(3) $$E_{t}=\frac{c\left(t\right)}{\int_{0}^{\infty }c\left(t\right)\cdot dt}$$

*E_t_* can be regarded as a probability function describing the probability of a tracer particle exiting the powder feeding system of the tablet press after entry of the whole tracer amount at one point of time (*t* = 0). A useful description of residence time functions can be achieved by determining their first and second momentum [35]. The first momentum is called the mean residence time *τ* and can be calculated by weighting the probability function with the time:

(4) $$\tau =\int_{0}^{\infty }t\cdot E_{t}\cdot dt$$

The mean residence time *τ* describes the average time a tracer particle needs to travel through the investigated system. For rotary tablet presses, the mean residence time heavily depends on the powder flow rate which is mainly influenced by the turret speed, number, and size of dies and the die filling efficiency.

The second momentum of a residence time distribution is its variance $\sigma_{t}^{2}$. The variance can be used to compare the system with two commonly used model systems: plug flow reactor (PFR) with only axial movement and no backmixing ($\sigma_{t}^{2}$ = 0) and continuous flow stirred tank reactor (CSTR), which describes a perfectly homogenous mixing of particles and corresponds with high $\sigma_{t}^{2}$ values [35]:

(5) $$\sigma_{t}^{2}=\int_{0}^{\infty }{\left(t-\tau \right)}^{2}\cdot E_{t}\cdot dt$$

Derived $\sigma_{t}^{2}$ values can be used to evaluate the intermixing inside the feeding system of the rotary tablet press, whereas higher $\sigma_{t}^{2}$ values show enhanced mixing. 

As described by several other authors [1,6,11,36], the powder flow rate in rotary tablet presses depends on material properties as well as on several process parameters, e.g. paddle speed, turret speed and filling depth. For a given experimental setup, the powder flow rate crucially affects the mean residence time (exemplarily shown in Figure 3a). Increasing powder flow rates correlate with decreasing mean residence times, while it increases if the powder flow rate is reduced. Therefore, to compare RTDs for different process parameters resulting in variations of powder flow rate, the RTDs have to be normalized by calculating the normalized time *θ*:

(6) $$\theta =\frac{t}{\tau }\;$$

The normalized residence time function *E_θ_* can then be determined:

(7) $$E_{\theta }=E_{t}\cdot \tau$$

By using the normalized residence time function over the normalized time, different process setups can be better compared and the influence of process parameters on RTDs can be evaluated (Figure 3b). 

To evaluate the influence of process parameters on the intermixing in the feed frame independent of the powder flow rate, the normalized variance *σ^2^* of the normalized probability function is calculated:

(8) $$\sigma^{2}=\frac{\sigma_{t}^{2}}{\tau^{2}}$$

Additionally, the mean residence time can be compared to the ideal filling time *t_f_* (s), which represents the required time to fill the volume of the feed system *V_R_* (m^3^) for a given volumetric flow rate $\dot{V}$ (m^3^ s^−1^). Assuming no dead volumes and a constant density $\rho_{b}$ (kg m^−3^), the mass flow $\dot{m}$ (kg s^−1^), estimated by the mass of sampled tablets, can be utilized to calculate *t_f_*:

(9) $$t_{f}=\frac{V_{R}}{\dot{V}}=\frac{V_{R}\cdot \rho_{b}}{\dot{m}}$$

Differences between the mean residence time and the ideal filling time can be used to evaluate the filling level inside the feeding system of the investigated rotary tablet press and thus, give useful insights into the influence of process parameters on the powder flow behavior in tablet presses.

## 3. Results and Discussion

### 3.1. Impact of Dyeing Process on Powder Properties

**Figure 4:**
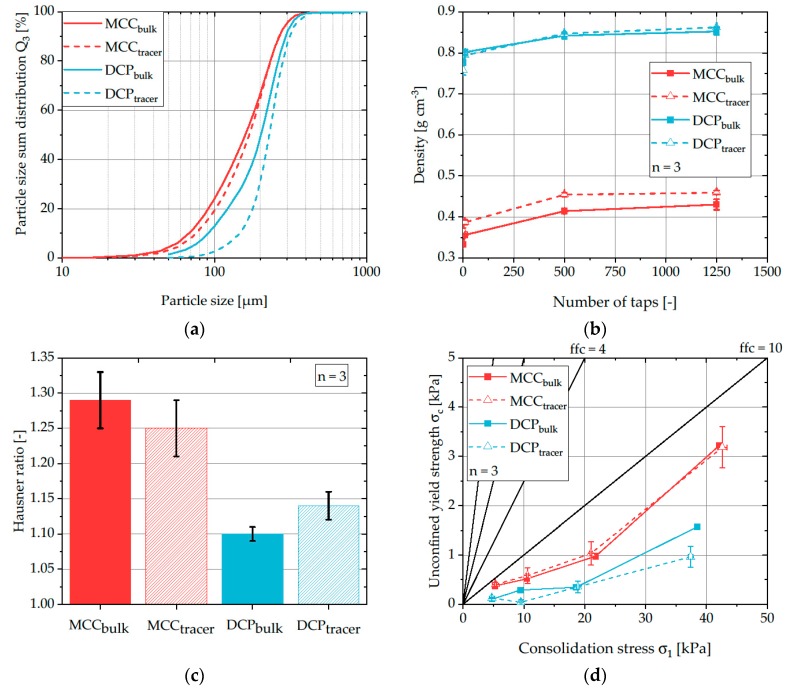
Particle size sum distributions (**a**), bulk and tapped densities (**b**), Hausner ratios (**c**), and ring shear cell results (**d**) of bulk and tracer powder. Symbols represent mean and error bars standard deviation.

To analyze the impact of process parameters on the RTD of excipients inside the feeding system of rotary tablet presses, the applied tracer has to exhibit comparable particle and powder flow properties as the investigated excipient to prevent segregation and unequal distribution. The powder flow behavior depends on several particulate properties, for example, the average particle size, the particle size distribution, the bulk and tapped densities and the cohesive forces acting between the particles. Therefore, those particulate properties were measured before and after the dyeing process to evaluate and estimate the influence of this process step on the powder flow behavior (Figure 4).

Particle size distributions of both bulk powders revealed that DCP has slightly higher particle sizes with x_50_ = 200 µm compared to MCC with x_50_ = 150 µm. For both materials, the particle size distributions of bulk and tracer powder are comparable and show only a minor shift towards larger particle sizes (Figure 4a) which could be attributed to the removal of fines and their deposition on the filter cartridges in the fluidized bed granulator at the beginning of the dyeing process.

The bulk and tapped densities show distinctive differences between both materials (Figure 4b). DCP exhibits higher bulk and tapped densities and a decreased tendency to reduce its volume. Thereby, according to the Hausner ratio classification, the material is expected to exhibit a good to excellent powder flow behavior. In contrast, MCC is more susceptible to compression during the tapping experiments as indicated by the increased *HR* values (Figure 4c). According to the classification, MCC is expected to show passable powder flow behavior. 

For DCP no significant differences in density are obtained for samples taken before and after the dyeing process. However, the resulting *HR* values of DCP slightly increase for dyed powder material. This difference is small compared to the difference between both materials and thus, is considered negligible. In contrast, the bulk and tapped density of MCC slightly increases after the dyeing process. However, this difference is only small and the variability of the resulting *HR* values show no significant differences between the bulk and tracer powder. 

Ring shear cell experiments revealed a superior flowability of DCP when compared with MCC and no significant influence of the dyeing process on the flowability of both materials (Figure 4d). Thus, as indicated by both Hausner ratio and flowability, two main conclusions could be drawn: On the one hand, it could be shown that DCP has the better flow behavior compared to MCC and on the other hand, the dyeing process does not substantially change the flow properties of the materials. 

Therefore, we concluded that the experimental setup is suitable to produce tracer with comparable flow properties to investigate the RTD of excipients inside rotary tablet presses. Additionally, the applied materials and their differences in powder flow properties can be used to differentiate the residence time behavior of free-flowing and cohesive powders in rotary tablet presses.

### 3.2. Estimation of Filling Level inside the Feed Frame of the XL 100

**Figure 5:**
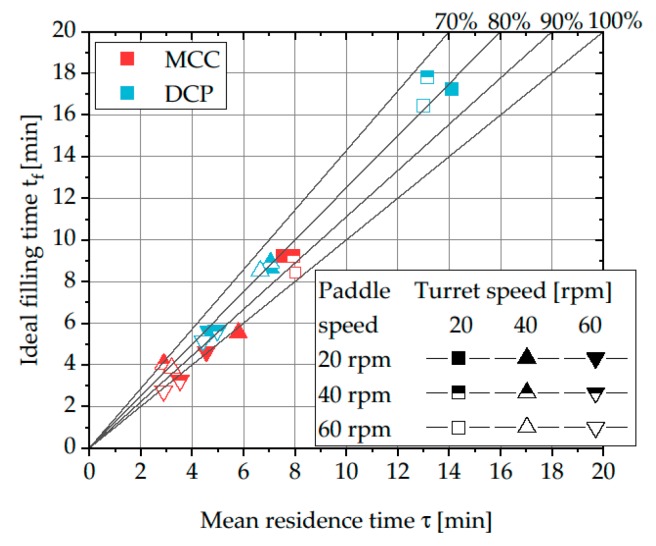
Comparison of mean residence time and ideal filling time for MCC and DCP using the rotary tablet press XL 100 and different levels of turret and paddle speed. Plotted lines represent different theoretical filling levels of the feed frame.

The calculated ideal filling time and measured mean residence time of each individual set of process parameters applied on the XL 100 were compared to estimate the filling level inside the feed frame (Figure 5). In the context of this consideration, only the filling degree in the feed frame was estimated by assuming that the filling pipe is filled and the powder contained in it exhibits the bulk density. Due to the complex geometry, the ideal filling time inside the feed frame of the XL 400 cannot be reasonably calculated and thus, no comparison of the mean residence time and the ideal filling time for the production press was conducted.

**Figure 6:**
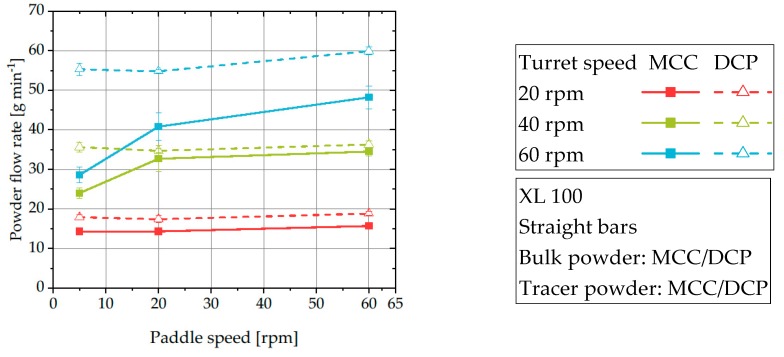
Powder flow rates of DCP and MCC using the XL 100 for three different levels of turret and paddle speed.

The comparison of the mean residence time and the ideal filling time allows the estimation of the filling level and thereby, the active volume of the powder feeding system. To illustrate the different levels of incomplete filling and thus, decreased active volume, slopes for filling levels of 70%, 80%, 90%, and 100% are added to the plot (Figure 5). For the majority of investigated process set-ups, the measured mean residence time is smaller than the ideal filling time, indicating a reduced active volume or a decreased bulk density of the powder inside the feed frame. In the context of this work, no systematic influence of process parameters on the deviation of mean residence time and ideal filling time was found. Studies of Dühlmeyer et al. and Peeters et al. showed that the filling level inside feed frames depends on the applied process parameters and material characteristics and varying filling levels in feed frames are common [1,37,38]. Based on the findings of Dühlmeyer et al. it is assumed that due to the dynamic process conditions, parts of the feed frame always remain unfilled. However, it could be found that DCP shows a stronger tendency to deviate from a complete filling level. This could be caused by the improved powder flowability resulting in an increased powder flow rate (Figure 6) which prevents powder from accumulating in the feed frame. These results are in good accordance with the findings of Dühlmeyer et al. who found also an increased filling level for material with higher bulk density [38]. As MCC showed a poorer flowability, it could be hypothesized that it is more likely to accumulate in the feed frame resulting in a better agreement between mean residence time and ideal filling time. Alternatively, MCC could tend to be more easily compressed in the feed frame and thus, exhibiting an increased bulk density corresponding with a better agreement of ideal filling time and mean residence time.

For future studies, it would be of high interest to further evaluate the influence of material and process parameters on the filling level inside rotary tablet presses. Thereby, this method represents an easy and quick approach to estimate the active volume inside the feed frame during the tableting process. 

### 3.3. Impact of Turret and Paddle Speed on RTDs

**Figure 7:**
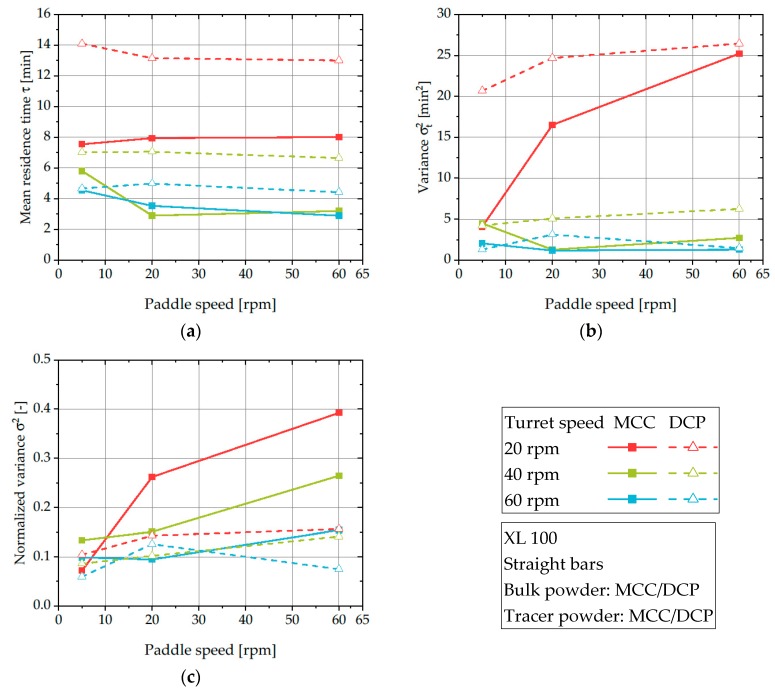
Influence of turret and paddle speed on mean residence time (**a**), variance (**b**), and normalized variance (**c**) of DCP and MCC using the XL 100.

As previously discussed, only the process parameters can be changed to control the flow behavior and thus, the resulting RTD for a fixed formulation. In this chapter, the influence of turret and paddle speed on RTDs of MCC and DCP is discussed. The turret speed directly determines the number of produced tablets per minute and thus, the amount of powder flowing through the feed frame at a given time. As a result, the RTD of the powder should be modified for increasing turret speeds and thus, powder flow rates. For both materials, an increase of the turret speed corresponds to a shortened average residence time (Figure 7a). 

DCP exhibits higher average residence times compared to MCC when the same process parameters were applied. This is caused by a considerably higher bulk density of DCP (0.777 g cm^−3^) compared to MCC (0.333 g cm^−3^). The tracer is placed at the same location inside the feeding pipe regardless of the investigated material and, by that, needs to pass the same volume before the withdrawal. However, the mass of DCP in this volume is much higher and, as approximately the same mass flow rate is applied by keeping the same target tablet masses, the reduced volume flow rate of DCP results in increased mean residence times. 

The mean residence time of DCP is virtually independent of the paddle speed at a given turret speed (Figure 7a). In contrast, the mean residence time of MCC slightly decreases for turret speeds of 40 and 60 rpm when the paddle speed is increased. The observed effect that MCC is more sensitive to paddle speed is because for unfavorable combinations (high turret speed with low paddle speed) the powder flow into the dies is reduced (Figure 6). As a result, the powder needs more time to flow through the feeding system resulting in an increased mean residence time. In contrast, the powder flow rate of DCP is virtually independent on the applied paddle speed (Figure 6) leading to a virtually constant mean residence time for a given turret speed. These results of powder flow rates are in good accordance with findings by Peeters et al., who found a significant impact of paddle speed on the tablet weight for MCC, but only a limited effect for DCP [1]. 

However, in the context of RTDs Dülle et al. found no influence of paddle speed on the mean residence time of the same grade of MCC [28]. These different trends could be related to the fact that Dülle et al. mixed MCC with a flow agent and the different structure of the applied feed frames. When no flow agent is added, the distinct differences found for both substances can be explained by their different powder flow properties. These results highlight the importance of considering powder mass flow when comparing the mean residence time to derive meaningful results and the influence of material properties on RTDs. 

The variance $\sigma_{t}^{2}$ describes the width of the RTD and can be used to characterize the intermixing inside the feed frame. Small $\sigma_{t}^{2}$-values are related to plug flow behavior and limited intermixing, while high values are characteristic for increased intermixing inside the investigated system comparable to a CTSR. For both materials, the variance decreases with increasing turret speed (Figure 7b) which is in good agreement with findings of Dülle et al., who found a decrease of both mean residence time and variance with rising powder flow rates for MCC mixed with a flow agent [27,28]. For DCP, a slight increase in the variance for increasing paddle speeds can be seen which is likely caused by increased intermixing. This trend of increased variance for higher paddle speeds was also observed by other researchers [27,28]. In contrast, the interplay of turret speed and paddle speed leads to different trends when applying MCC. For low speeds, a significant increase in the variance is seen with increasing paddle speed, while for higher turret speeds (40 and 60 rpm) the overlap of increased powder flow and intermixing does not lead to a clear trend. These results clearly show that a meaningful interpretation of the RTDs and the estimation of intermixing for different powder flow rates require a mass flow rate independent consideration. This limitation can be overcome by calculating the normalized variance *σ^2^* (Figure 7c).

For DCP, no systematic influence of turret speed or paddle speed on *σ^2^* is found. Additionally, the calculated normalized variance values range between 0.05–0.15 which can be seen as an indicator for plug flow behavior inside the feed frame [35]. Therefore, it can be assumed that for a given powder flow rate, the powder flow behavior of DCP is virtually independent of the applied process parameters for the investigated rotary tablet press. Furthermore, significantly higher normalized variance values were observed for MCC (0.1–0.4). It can, therefore, be assumed that the flow behavior of MCC is more influenced by the process parameters and tends to show more intermixing inside the feed frame. This difference to DCP can be attributed to the higher cohesion and thus, the poorer flow properties, which causes MCC to stronger deviate from an ideal plug flow. In terms of stress distributions to the powder inside the feed frame as well as the continuous production of tablets, narrow RTDs are favored. Thereby, it can be proved that fairly flowing powders are more prone to show high deviations from plug flow behavior and thus, higher residence time variance inside the feed frame.

### 3.4. Influence of Tracer Properties on RTDs 

The previously presented experiments used tracer material of comparable particulate properties as the excipients used and thus, can be used to describe the residence time behavior of the investigated excipient itself. However, commercial formulations typically consist of blends of several compounds with different particulate properties. Therefore, it is interesting to check for the influence of varying, defined particulate properties on the RTD inside the feed frame, by that also elucidating the propensity of segregation. For this purpose, the effect of MCC and DCP as tracer material within bulk material of DCP was studied and the obtained RTDs are compared.

**Figure 8:**
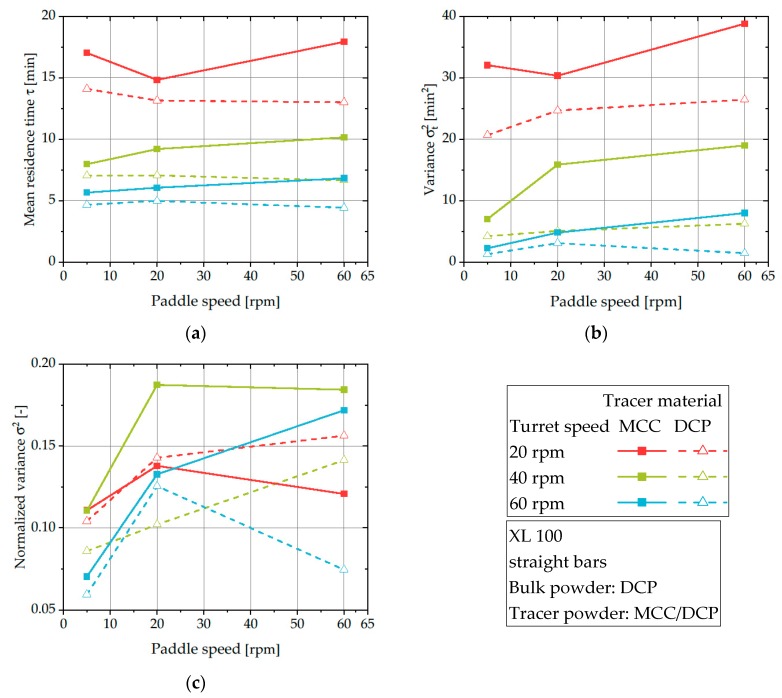
Mean residence time (**a**), variance (**b**), and normalized variance (**c**) of DCP and MCC acting as tracer material in a DCP bulk on the XL 100 for different levels of turret and paddle speed.

It is evident from Figure 8a,b that for all investigated process parameters in this setup, MCC as a tracer exhibits longer mean residence time and variance values when compared with DCP as a tracer. As the powder flow rate of DCP was shown to be independent of the applied paddle speed and the small volume of applied tracer should have no significant influence on the bulk density, the prolonged mean residence time and variance of MCC cannot be explained by a divergent powder flow rate of the bulk powder. A possible explanation for the prolonged mean residence time could be the different flowability of both materials. MCC compared to DCP has a poorer flowability and could, therefore, tend to stay longer in the feed frame as DCP is more easily transported by the feeding system. Additionally, it was visually observed that MCC tends to stick on stainless steel surfaces possibly due to increased adhesive properties. This sticking could result in an increased residence time inside the feed frame. As an additional mechanism, the segregation of MCC could take place. Due to the significantly lower bulk and solid density, the selective segregation of MCC can result in the favored flow of DCP into the dies and thus, the increased residence times of MCC in the feed frame.

For all applied process parameters, $\sigma_{t}^{2}$ values of MCC as a tracer are systematically higher, correlated with an enhanced intermixing (Figure 8b). An increase in the paddle speed results in higher variance values. This trend is most likely caused by the different flow properties of the materials caused by different interparticulate properties. As shown before (Figure 7c), MCC seems to be more susceptible to being mixed and, thereby, the deviation from an ideal plug flow behavior is also increased in the case of DCP as a bulk powder. Additionally, the increased mean residence time of DCP (Figure 7b), caused by its higher bulk density, extends the general residence time of MCC inside the feed frame and, thus, the variance values. The fact that this trend is also visible for MCC as a tracer in the free-flowing bulk of DCP shows that the cohesiveness of the investigated powder component can have a significant effect on its RTD, result in higher intermixing, even in a free-flowing bulk material, and can lead to RTD-wise identification of segregation tendencies.

The trend of increased normalized variance for MCC (Figure 8c) confirms the previous findings of deviating powder flow behavior. Since the normalized variance can be used to estimate the powder flow behavior in relation to a PFR independent on the powder flow rate, it can be seen that MCC shows a higher tendency to deviate from ideal PFR flow behavior. As discussed before, these deviations are unfavorable for continuous production as well as consistent tablet properties.

In general, the observed differences highlight that even small variations in particulate properties (as MCC has a comparable particle size to DCP and differs intermediately in flowability and strongly in bulk density) can result in considerable differences of residence time inside the rotary tablet press. Probably this results in varying values of stress applied to the material and of segregation, which results in undesired tablet property alteration over time.

### 3.5. Transferability of RTDs Results Between Different Scales of Rotary Tablet Presses

During the development of new drugs, a scale-up of the tableting process from lab to production scale is necessary to ensure the time- and cost-efficient production. Consequently, it is interesting to compare the RTD of powder in rotary tablet presses of different scales. Therefore, residence time experiments with DCP are conducted using the production-scale tablet press XL 400 with a comparable experimental setup. In contrast to the XL 100, the feed frame of the XL 400 consists of dosing and a metering chamber each equipped with a paddle. For the XL 400, also the effect of different geometries of the dosing paddle was evaluated.

**Figure 9:**
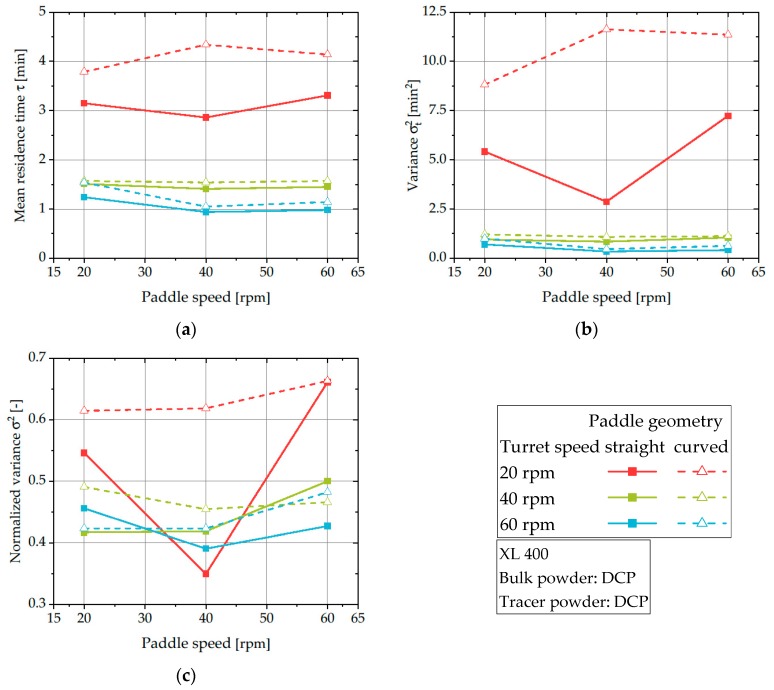
Mean residence time (**a**), variance (**b**), and normalized variance (**c**) of DCP for process parameters using the XL 400.

Figure 9a displays the mean residence time of DCP in the feed frame of the XL 400. Generally, the increase of the turret speed results in a significant drop in the mean residence time. Interestingly, the use of curved bars as dosing paddle results in an increase of the mean residence time for all applied experimental set-ups. This could be related to an increased intermixing of the tracer inside the feed frame for curved paddles as indicated by Figure 9b,c. Generally, the variance increases when curved paddles are employed, especially visible for the slowest turret speed. The reason for the enhanced intermixing of curved paddles could be assigned to the increased clearance between the bars of the dosing paddle and the bottom plate of the feed frame. An increased clearance supports the transport of powder between the interpaddle volumes divided by the bars and thus, amplifies the intermixing inside the feed frame. In contrast, the dosing paddle with straight bars could entrap the powder between its bars, effectively reducing the intermixing and thus, narrowing the RTDs. Similar trends were found by Dülle et al. for MCC using a Fill-O-Matic in a Fette 102i [30], indicating that this effect is generally detectable for several materials.

**Figure 10:**
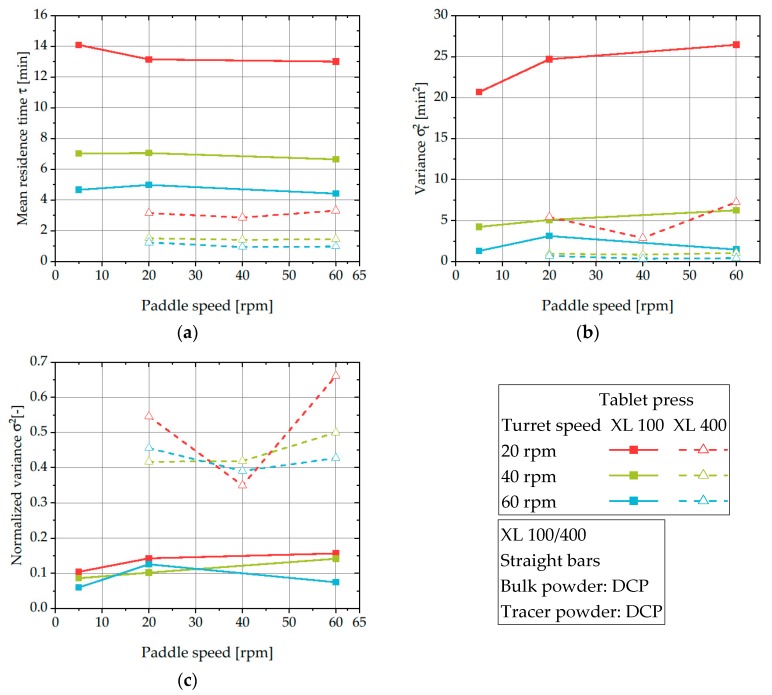
Mean residence time (**a**), variance (**b**), and normalized variance (**c**) of DCP on different scales of rotary tablets for varying process parameters.

When comparing the obtained RTDs and derived parameters *τ*, $\sigma_{t}^{2}$, and *σ^2^* for different tablet presses, it is obvious, that the mean residence time and variance considerably decrease when the XL 400 is applied (Figure 10). 

**Figure 11:**
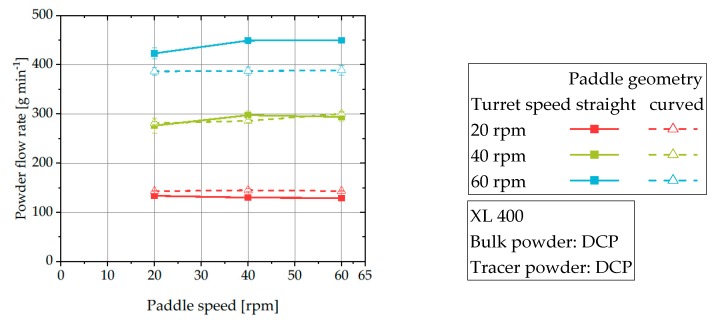
Powder flow rate of DCP at XL 400 for different process parameters.

This is partially caused by the different entrance point of the tracer into the rotary tablet presses. In the case of the XL 100, the powder has to pass the filling pipe to enter the feed frame and thus, longer residence times are measured. Besides this effect, the XL 400 exhibits an increased powder flow rate (compare Figure 6 and Figure 11) which effectively reduces the residence time by accelerating the powder transfer through the feed frame. By this, the mean residence time of ~13 min (XL 100, turret speed of 20 rpm) is reduced to ~1 min (XL 400, turret speed of 60 rpm). As the residence time inside the feed frame is directly linked to the stress applied to the processed powder, this has to be considered during the scale-up of the tableting process. Those differences in applied stress inside the feed frame are crucial and possibly deteriorate the overall tablet quality, e.g., by overlubrication caused by the shear stress inside the feed frame. However, the applied experimental setup limits the comparability of different tablet presses due to the different powder flow rates which heavily influence the RTDs.

**Figure 12:**
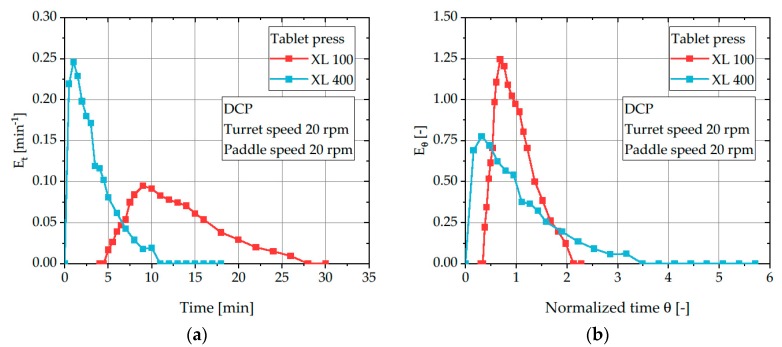
Residence time distribution *E_t_* (**a**) and normalized residence time distribution *E_θ_* (**b**) of DCP for turret and paddle speeds of 20 rpm on different tablet presses.

In contrast to the reduced mean residence time and variance, the normalized variance is mass flow independent and can be used to compare both rotary tablet presses and assign them to one of the model systems (PFR or CTSR). The *σ^2^* values significantly differ between both rotary tablet presses. While the XL 100 has comparatively low values of 0.05–0.15 and thus behaves more like a PFR, much higher values in the range of 0.35–0.65 were observed for the XL 400 indicating a much stronger mixing effect and behavior in the direction towards a CTSR. This effect is especially visible when comparing the obtained RTDs of DCP on both investigated rotary tablet presses (Figure 12). After normalization, the RTD of DCP in the XL 100 is considerably narrower, highlighting a powder flow behavior comparable to a PFR. In contrast, DCP exhibits substantially wider RTDs in the XL 400 and thus, shows increased intermixing. The increased intermixing inside the feed frame of the XL 400 is possibly caused by the increased hold-up volume as well as the presence of a metering chamber with a second paddle. 

It is hypothesized that the powder that enters the metering chamber during the backdosing remains inside the metering chamber for a considerable amount of time before it is eventually transported back into the feeding chamber. As discussed before, these prolonged residence times can have a deteriorating effect on the powder properties and thus, are to be avoided. Therefore, in terms of RTDs, single chamber feed frames are desirable to avoid the separation of material fractions which can result in high differences in residence time and thus, inhomogeneity of tablet properties. These results highlight the differences of feed frames of varying scales of tablet presses and emphasizes the challenge of scaling-up the tableting process on different scaled rotary tablet presses.

## 4. Conclusions

A universal method to produce tracer powders with effectively equal particle and bulk properties as the investigated bulk powder was developed. The evaluation of the RTDs, mean residence time *τ*, variance $\sigma_{t}^{2}$, and normalized variance *σ^2^* of two common excipients with different powder flow properties (MCC and DCP) was performed on different scales of rotary tablet presses (pilot-scaled XL 100 and production scaled XL 400) at different turret and paddle speeds. The mean residence time was used in a facile method to estimate the degree of filling in the feed frame of the XL 100 and it was observed to be incomplete for the majority of the tests. For all experiments, a strong influence of the powder flow rate on the mean residence time and variance was found, as expected. Therefore, the mass flow-independent normalized variance was used to estimate the intermixing in the feed frame. RTDs of the free-flowing DCP are virtually unaffected by process parameters, while an increased intermixing for higher paddle speeds was found for the poorer flowing MCC. Studies applying the worst and the best flowing component of a tableting formulation as tracers in the common bulk powder provide a method to estimate the susceptibility of such powder blends towards segregation and selective overlubrication.

Comparing the rotary press scales, the pilot-scale showed narrower RTDs and behaved more like a PFR, while the wider RTDs of production scale indicate a stronger intermixing and a more CTSR-like behavior. Such an intermixing and smoothing effect on alterations of API concentration can intendedly be applied to improve content uniformity, i.e., in continuous process chains. In contrast to that, high intermixing and a broad RTD may be unfavorable for shear sensitive blends such as highly lubricated powder blends. This again stresses the fact, that the formulation and the process (equipment and parameters) must be developed and deliberately selected in coordination with each other. This work adds to the process understanding which is necessary for such rational decisions in future development. Future studies will focus on the impact of equipment connected to the feed frame (e.g., filling chutes) on the RTD to deepen the knowledge obtained.

The results of this work can be used for the further development of continuous production and clearly show the influence of material parameters on the powder flow behavior inside rotary tablet presses. Furthermore, the applied experimental setup can be used to roughly estimate the filling level of the feed frame as well as help to identify possible challenges during the scale-up of tableting processes.

## Figures and Tables

**Table 1:** Process parameters of residence time distribution experiments at XL 100 and XL 400.

Tablet Press	Turret Speed (rpm)	Paddle Speed of Dosing Paddle (rpm)	Paddle Geometry of Dosing Paddle
XL 100	20, 40, 60	5, 20, 60	Rectangular, straight
XL 400	20, 40, 60	20, 40, 60	Rectangular, curved
			Round, curved

**Table 2:** Applied machine settings for RTD experiments on XL 100 and XL 400.

Tablet Press	Material	Filling Height (mm)	Dosing Height (mm)
XL 100	MCC	14	10
	DCP	8	5
XL 400	DCP	14	9

## Supplementary Materials

The following are available online at https://www.mdpi.com/1999-4923/12/3/283/s1, Table S1: Overview of applied experimental set ups, Table S2: Overall run duration of RTD experiments for applied experimental set ups.
