# Supplementary material for: Impact of Particle and Equipment Properties on Residence Time Distribution of Pharmaceutical Excipients in Rotary Tablet Presses
Source: Pharmaceutics. 2020 Mar 21;12(3):283. doi: 10.3390/pharmaceutics12030283 (PMC7151020; doi:10.3390/pharmaceutics12030283)
Supplement: Supplementary file 1 [file pharmaceutics-12-00283-s001.pdf]

pharmaceutics

MDPI

Supplementary Materials: Impact of Particle and Equipment Properties on Residence Time Distribution of Pharmaceutical Excipients in Rotary Tablet Presses

Table S1: Overview of applied experimental set ups.

Tablet Press	Bulk Material	Tracer Material	Dosing Paddle Geometry	Turret Speed [rpm]	Paddle Speed [rpm]
					5
				20	20
					60
					5
	MCC	MCC	Rectangular, straight	40	20
					60
					5
				60	20
					60
					5
				20	20
					60
					5
XL 100		DCP	Rectangular, straight	40	20
					60
					5
				60	20
	DCP				60
					5
				20	20
					60
					5
		MCC	Rectangular, straight	40	20
					60
					5
				60	20
					60
					20
				20	40
					60
					20
			Rectangular, curved	40	40
					60
					20
				60	40
XL 400	DCP	DCP			60
					20
				20	40
					60
					20
			Round, curved	40	40
					60
					20
				60	40
					60

Table S2. Overall run duration of RTD experiments for applied experimental set ups.

Tablet Press	Bulk Material	Turret Speed [rpm]	Run Duration [min]
XL 100	DCP	20	26
		40	19
		60	16
	MCC	20	19
		40	19
		60	11.5
XL 400	DCP	20	18
		40	9.83
		60	5.45
